# Metagenomic next-generation sequencing assists in diagnosing *Pneumocystis Jirovecii* pneumonia in non-HIV patients: a case report

**DOI:** 10.1016/j.rmcr.2025.102289

**Published:** 2025-09-18

**Authors:** Ying Zhang, Anbao Chen, Chunyan Yang, Li Guan, Chun Wang

**Affiliations:** a Department of Emergency Intensive Care Unit, Second Affiliated Hospital of Kunming Medical University, Kunming, China; b Department of Emergency, Second Affiliated Hospital of Kunming Medical University, Kunming, China

**Keywords:** Pneumocystis jirovecii pneumonia, Metagenomic next-generation sequencing, Non-HIV, Diabetic ketoacidosis, Diagnosis

## Abstract

**Background:**

*Pneumocystis jirovecii* Pneumonia (PJP) is a pulmonary opportunistic fungal infection with an incompletely elucidated pathogenesis. In recent years, non-human immunodeficiency virus (HIV) -infected PJP patients have exhibited rapid progression, poor prognosis, and a greater mortality rate compared to their HIV equivalents, necessitating timely detection and management, which are both critical and problematic.

**Case report:**

We report a young patient admitted with diabetic ketoacidosis characterized by rapidly progressing acute respiratory failure with negative pathogen blood cultures, serum antibodies and polymerase chain reaction results, and a normal CD4^+^ lymphocyte count. Anti-HIV antibody were negative. A computed tomography scan of the chest revealed patchy opacities in both lower lungs, a nonspecific manifestation. However, metagenomic next-generation sequencing (mNGS) of bronchoalveolar lavage fluid detected high *Pneumocystis jiroveci* sequence counts and a markedly elevated 1,3-β-D-glucan test titer. Following the diagnosis of non-HIV-infected PJP, the patient was discharged after 13 days with a positive outcome, attained through systematic management involving Trimethoprim-sulfamethoxazole anti-infective medication and stringent glycemic control.

**Conclusion:**

Insufficient glucose management may be an important susceptibility factor for immunocompetent persons with non-HIV-infected PJP patients. MNGS serves as an effective method for rapid diagnosis and medication adjustment when signs, symptoms, and imaging findings of PJP are nonspecific.

## 1 Introduction

*Pneumocystis jirovecii* pneumonia (PJP) is an opportunistic respiratory fungal illness caused by *Pneumocystis jirovecii* (PJ), with a significant rise in incidence and frequency observed in recent years. Commonly appears in patients with human immunodeficiency virus (HIV) infection, malignant hematologic neoplasms, solid tumors, organ failure, extended immunosuppressive treatment, and hormone administration. The mortality of HIV-positive patients with severe PJP in the intensive care unit (ICU) was 28.5 % [1]. Still, for non-HIV-infected PJP patients, it was 49.7 % [1]-75.6 % [2], escalating with the abrupt onset of respiratory failure and a postponed diagnosis of PJP [3]. Furthermore, non-HIV-infected PJP patients have complex infections, atypical clinical symptoms, rapid progression, and high mortality, so accurate diagnosis and timely intervention are more urgent [4]. The majority of the literature discusses risk factors and treatment strategies for immunocompromised non-HIV-infected individuals, but data concerning immunocompetent patients are scarce.

We report a case of a non-HIV-infected PJP patient who had no previous illnesses and was admitted solely for diabetic ketoacidosis (DKA). The clinical presentation was non-specific, yet rapid identification of a high sequence count of *Pneumocystis jirovecii* was achieved through bronchoalveolar lavage fluid (BALF)-metagenomic next-generation sequencing (mNGS) followed by antibiotic regimen modification and favorable clinical outcomes. This has prompted further consider of the potential role of inadequate glucose regulation as a significant risk factor for immunocompetent non-HIV-infected PJP patients, highlighting the necessity for physicians to emphasize rapid detection, early diagnosis, and treatment of nonspecific fungal infections.

## 2 Case report

**Fig. 1:**
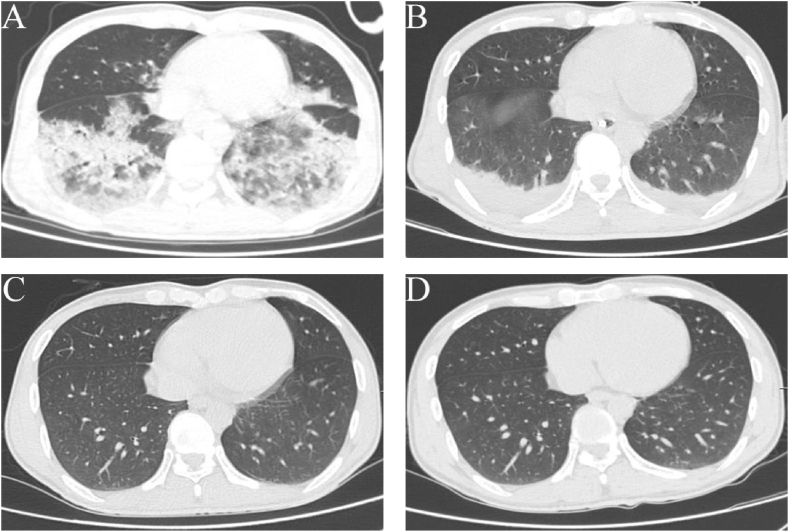
Chest CT imaging of the patient. A. Chest CT scan at the time of presentation revealed diffuse patchy shadows in both lungs; B. A chest CT scan prior to tracheal intubation and extubation indicated that the patchy shadows in both lungs had greatly diminished; C. A chest CT scan prior to the patient's hospital discharge revealed that the patchy shadows in both lungs had significantly improved; D. A chest CT scan one month later revealed that there were no significant patchy shadows in either lung. CT, computed tomography.

This 35-year-old male was initially diagnosed with DKA at a local clinic, presenting with polyuria, polydipsia, excessive thirst, a fluid intake of approximately 7500 ml per day, and a weight loss of approximately 8 kg per month. He received rehydration and acid-base corrective therapy for seven days, the antibiotic treatment of which is unclear. He was transferred to our hospital due to progressing cyanosis, altered consciousness, dyspnea, and wheezing. The initial laboratory findings revealed the white blood cell count was 14.05 × 10^9^/L (3.50–9.50), the neutrophil ratio was 86.40 % (40.0–75.0), the platelets were 187 × 10^9^/L (125–350), pH was 7.091, ABE was −25.1mmol/l, HCO^3−^ was 2.6mmol/l, PRO-BNP was 383pg/ml, IL-6 was 45.20 pg/m, PCT was 1.83 ng/ml, CRP was 47.22 mg/l, LDH was 328 U/L, anti-HIV antibody was negative. Normalcy CD4^+^ lymphocyte counts were 780 cells/μL, and a normal CD4/CD8 (CD4/CD8, 2.10) was also observed. A chest computed tomography (CT) scan revealed diffuse opacities and patchy consolidations in both lungs (Fig. 1A). He received empirical treatment for severe community-acquired pneumonia with broad-spectrum antibiotics, including cefoperazone sodium sulbactam sodium (3.0g, once), and moxifloxacin hydrochloride injection (0.4g:2.0g, once). However, his condition deteriorated due to acute respiratory distress syndrome (ARDS), circulatory failure, and multiorgan failure. The patient was intubated with ventilator-assisted breathing, subsequently underwent bedside fiberoptic bronchoscopy and alveolar lavage, with the bronchoalveolar lavage fluid remained for metagenomic next-generation sequencing, culture, PCR, and BDG testing before being transferred to our department for further therapy. And empirical antibiotics were adjusted to meropenem (1000 mg IV every 8 h). His family stated that he was well and did not recall being in contact with ill persons or animals in the weeks before this admission.

BALF-mNGS identified sequences of *Pneumocystis jirovecii* with 773 reads and *Klebsiella pneumoniae* with one read. Trimethoprim-sulfamethoxazole (TMP-SMX) was added (at a dose of TMP16 mg/kg/day and SMX 80 mg/kg/day [5]). The following evaluation revealed negative results for BALF polymerase chain reaction (PCR), serum antibodies, cultures from respiratory and blood specimens, serum BDG and GM tests in addition to 1,3-β-D-glucan (BDG) assays of BALF suggesting 449.8 pg/mL. The patient remarkably recovered by implementing stringent glycemic regulation, antibacterial treatment, fluid management, and physiotherapy. He was extubated on the fifth day of hospitalization. Table 1 displays the findings of his arterial blood gas analysis showed the oxygenation index is gradually recovering. Following the resolution of the CT-identified exudative opacities in the lungs (Fig. 1B), the patient was extubated, recovered consciousness, and discharged after 13 days, with the CT scan revealing almost no exudative shadows in both lower lungs (Fig. 1C). One month later, the patient underwent evaluation and had no breathing problems, while a subsequent CT was conducted (Fig. 1D), confirming the significant recovery. Simultaneously, the patient underwent BALF-mNGS again, which did not identify any pathogens.

**Table 1:** 

	D0(ER)	D1	D2	D3	D4	D5	D6	D7
PH	7.091	7.557	7.486	7.551	7.499	7.434	7.457	7.439
pCO2	15	28.4	29.2	26.6	26.9	27.8	33.4	30.6
pO2	87	115	118	113	119	121	106	100
pO2/FIO2	175	185	235	245	298	292	292	355

## 3 Discussion

PJP is common in people with congenital or acquired immunodeficiencies, with advances in medical technology, an increasing number of immunocompromised non-HIV-infected patients, especially with the prevalence of organ transplants, the increase in malignant tumors, autoimmune diseases, and the widespread use of immunosuppressants and glucocorticosteroids, are becoming susceptible to PJ infection and are being detected [6].

In this case, this patient had no prior medical history, and the initial episode was DKA, indicating that blood glucose levels were not well monitored or managed. Identifying a significant prevalence of PJ in BALF, without any indication of immunocompromise, raises the question of whether inadequate glycemic management is a critical predisposing factor among immunocompetent individuals with PJP. Diabetes mellitus is recognized as a substantial risk factor for fungal lung disease [7]. The processes implicated may pertain to modified immune cell functionality, including diminished chemotaxis, phagocytosis, and bactericidal capacity of macrophages, compromised cellular immune control, and an acidic milieu conducive to fungal proliferation; the precise mechanisms remain unidentified [8]. This contrasts with PJP patients suffering from T-lymphocyte deficiency, particularly CD4^+^ T-lymphocytopenia, characterized by compromised macrophage function and adherence of alveolar epithelium, which obstructs gas exchange and significantly impacts host prognosis [9,10].

In recent years, the rapid development of molecular detection technology has led to the widespread application of mNGS in clinical practice due to its high throughput, speed, and accuracy, particularly in identifying co-pathogens of critical illnesses. Some research indicates that the prevalence of PJ among fungal sequences identified through second-generation sequencing exceeds 85 %, with BALF mNGS demonstrating enhanced sensitivity and specificity in diagnosing Pneumocystis jiroveci pneumonia in HIV-negative patients. A negative blood BDG outcome does not entirely exclude out the diagnosis of non-HIV PJP patients and should be evaluated in conjunction with, but not limited to, underlying variables that predispose BALF-mNGS offers considerable advantages [11,12], especially in this case of atypical presentation.

Regarding PJ colonization and infection,the question was likely considered with greater urgency during the diagnosis and medication adjustment phases in this case. Research indicates that PJ colonization rates in immunocompetent patients range from 0 % to 65 % [13]. The case presented no risk factors as documented in the literature, lacked a typical history, clinical presentation, and imaging changes, and showed no decrease in CD4^+^ T lymphocytes, elevated LDH, or detectable pathogens in blood cultures or serum antibodies. This absence of typical indicators led to an oversight regarding the association with PJ. However, the detection of *Pneumocystis japonicus* in the alveolar lavage fluid via mNGS prompted a reconsideration of the distinction between colonization and infection. The literature indicates that the optimal threshold for identifying PJ infection and colonization is 14 readings and a BDG of 88.6 pg/ml [14]. The elevated serial count of PJ in BALF-mNGS, along with the significant concentration of BDG test, prompted consideration of PJP as a critical diagnosis. A study involving 158 patients with PJP revealed that 94.9 % were hospitalized due to acute respiratory failure. The mortality rates were 31.6 %, 35.4 %, and 40.5 % for ICU, hospitalized, and patients at 6 months, respectively. Furthermore, the initiation of therapeutic antibiotics 96 hours post-ICU admission correlated with an increased rate of mortality, while the administration of corticosteroids for PJP treatment was linked to a heightened incidence of death [15], which makes us improve awareness of PJP and do early pathogenetic testing for TMP/SMX, which is the preferred medication for treating PJP; however, evidence on its efficacy in non-HIV populations is lacking [5]. Consequently, TMP-SMX was administered. Fortunately, the patient was successfully discharged, and BALF-mNGS follow-up did not detect any pathogens. This provided additional confirmation of the infection diagnosis.

## 4 Conclusion

Non-HIV-infected PJP is a prevalent lung infection in the ICU, with life-threatening situations and a dismal prognosis. In patients with nonspecific manifestations of immunocompetent PJP, inadequate glycemic control may serve as an important susceptibility factor. Timely diagnostic measures, including BALF-mNGS, specialized tissue staining, and PCR, should be used for precise diagnosis and treatment.

## CRediT authorship contribution statement

**Ying Zhang:** Writing – original draft. **Anbao Chen:** Formal analysis. **Chunyan Yang:** Resources. **Li Guan:** Investigation. **Chun Wang:** Writing – review & editing, Writing – original draft, Data curation, Conceptualization.

## Consent to participate

Informed consent was obtained from all individual participants included in the study.

## Consent to publish

The authors affirm that human research participants provided informed consent for publication of the images in Figures.

## CARE checklist (2016) statement

The authors have read the CARE Checklist (2016), and the manuscript was prepared and revised according to the CARE Checklist (2016).

## Funding

This study was supported by Yunnan Fundamental Research Project (grant NO.202501AT070261) and Doctoral Research Projects (grant NO.2025BS02).

## Declaration of competing interest

The authors declare that they have no known competing financial interests or personal relationships that could have appeared to influence the work reported in this paper.
